# Some notes on the use, concept and socio-political framing of ‘stigma’ focusing on an opioid-related public health crisis

**DOI:** 10.1186/s13011-020-00294-2

**Published:** 2020-08-03

**Authors:** Benedikt Fischer

**Affiliations:** 1 Schools of Population Health and Pharmacy, Faculty of Medical and Health Sciences, University of Auckland, 85 Park Rd, Grafton, Auckland, 1023 New Zealand; 2 Department of Psychiatry, University of Toronto, Toronto, Canada; 3 Centre for Applied Research in Mental Health & Addiction, Simon Fraser University, Vancouver, Canada; 4 Department of Psychiatry, Federal University of Sao Paulo (UNIFESP), Sao Paulo, Brazil

**Keywords:** Canada, Opioids, Policy, Public health, Stigma

## Abstract

Canada has been home to a longstanding public health crisis related to opioids, including an extensive mortality and morbidity toll in the face of substantive intervention gaps. Recently (2019), two extensive reports from preeminent federal authorities – the Chief Public Health Officer and the Mental Health Commission of Canada – have been tabled with detailed, core focus on the phenomenon of ‘stigma’ and its impacts on substance/opioid use and harms. The reports present extensive descriptions of the nature and effects, as well as a multitude of prescriptions for remedial measures and actions to “stop the cycle of stigma”. Closer reading of the documents, however, suggests substantial conceptual and empirical limitations in the characterization of the – multi-faceted and challenging – nature and workings of ‘stigma’ as a socio-political, structural or individual process or force, specifically as it applies to and negatively affects substance use and related outcomes, primarily the wellbeing of substance users. Concretely, it is unclear how the remedial actions proposed will materially alleviate stigma process and impacts, especially given apparent gaps in the issues examined, including essential strategies – for example, reform of drug user criminalization as a fundamental element and driver of structural stigma - for action that directly relate to the jurisdictions and privileged mandates of the report sources themselves as health and policy leaders. The commentary provides some concrete while subjective notes and observations on the dynamics of stigma as applies to and framed for substance/opioid use, as well as strategies and measures necessary to both tangibly address the material health and wellbeing of substance users, and related forces of stigma, in the distinct context of the opioid crisis in Canada.

## Background

Canada has been entrenched in an unprecedented public health crisis related to opioids for far more than a decade [1]. This crisis has taken an estimated 20–25,000 (mostly young or middle-aged) lives through opioid poisoning (overdose) fatalities over the most recent past 10 years, with > 4600 such deaths alone in 2018 [2, 3]. Even more such fatalities are, again, anticipated for 2020. For comparative illustration, the number of opioid-related deaths in Canada for the period 2016–19 (15,393) have far exceeded the number of COVID-related deaths (8687 by July 2020), and distinctly more so outside of older-age groups). A tangible, negative impact of opioid-related mortality on the general population’s life expectancy has been ascertained [4]. For years now, there have been fundamentally urgent and persistent calls (and correspondingly numerous promising statements) for expanded, better and overall more effective responses to the ongoing opioid-related public health crisis.

In 2019 – at a recent peak-point of opioid mortality – two prominent, federal-level reports, 1) The Chief Public Health Officer’s (CPHO) Report on the ‘State of Public Health in Canada’ [5], 2) the Mental Health Commission of Canada’s (MHCC) Report on ‘Stigma and the Opioid Crisis’ [6] were tabled and widely disseminated. Both these elaborate reports centrally focus on the role of ‘stigma’ for chronic disease and public health, and specifically ‘substance use’ and the ‘opioid crisis’ (the MHCC report with such exclusive focus, the CPHO report within a broader focus on stigma and public health). ‘Stigma’, at its foundations, is a sociological concept, crucially furthered by the Canadian-born sociologist Erving Goffman [7, 8]. Essentially, it indicates the ascription of negative attributes or assumptions (or ‘stereotypes’ or ‘mark of disgrace’) on a person because of certain properties or behaviours outside their control, and consequential loss in social status, opportunity, and care or support (including possible ‘discrimination’) [9].

Luoma provides some essential conceptual and practical characteristics of stigma. Stigma is produced and reproduced in many ways, including common ‘cultural practices’ of everyday life, and highly resistant to change. It can be helpful to distinguish between ‘organizational’ or structural level, and individual-level processes of stigma, both of which include (structural or individual, respectively) ‘prejudice’ and ‘discrimination’ as ways of enactment of stigma. For example, organizational/structural stigma, through related power processes, involves (intentional or un-intentional) policies or organizational rules, restrictions or opportunity barriers towards stigmatized group; whereas individual-level stigma can be divided into public (e.g., the individual reactions or judgments) about a stigmatized group (e.g., ‘addicts’) as well as ‘self-stigma’ (e.g., the internalization of negative self-value and status, and consequential self-preclusion from key opportunities (e.g., treatment) or negative outcomes experienced by the stigmatized individual themselves. A large variety of different strategies and approaches to reduce stigma have been identified and tried, with however limited demonstrated effects on reducing stigma [10].

‘Stigma’ has been given distinct (while limited, e.g., when compared to mental health) attention in the psychoactive substance use realm, in part also related to the conflicting underlying social concepts or explanations (e.g., crime versus moral failure versus disease models) of ‘addiction’, and consequential implications for the social identities, status and interventions geared at the user [9]. Luomo notes that research on stigma in the addiction realm is in its “infancy”, and that even less is known on “how to reduce stigma in this area.” [10]

## Two pre-eminent ‘anti-stigma’ manifests

Both above-mentioned reports ascribe fundamental and sweeping cause-effect agency, as well as necessary remedial prescriptions to the phenomenon of ‘stigma’ as applied to the current public health crisis of substance/opioid use in Canada. For example, the CPHO’s report lays out in elaborate detail [5]; pp.23–56] how ‘stigma’ creates a fundamental “us versus them” between substance users and society, resulting not only in “significant economic costs, barriers to housing, employment, health care, productivity loss”, functions as the root of “discrimination”, wrongfully blames substance users for “poor willpower”, and projects them to be “dangerous and reckless” and implies them to be not suffering from “real illness”. Beyond, stigma is listed as a driving factor of decreased service use, concealment of substance use, and health-harming coping behaviours (e.g., isolation, needle sharing), poorer health and quality-of-life (QOL), limited treatment uptake and poorer outcomes for substance users. Based on remedies prescribed for the stigma “cycle [to be] effectively stopped” and for “resisting the impacts of stigma” it is emphasized that required action need to occur on many (e.g., individual, institutional, population) levels, yet concretely by changing “biased and outdated language”, “strengthening resilience” (e.g., through education), and devising “cultural competence” interventions for health care providers towards the development of “awareness, knowledge and attitudes”. All the while it is then categorically acknowledged in the report that “it is difficult to know ‘what works’, in what context, to address stigma and discrimination” (p.53).

Similarly, the MHCC’s report [6] – including a related review paper from one of the authors as integrated elementary content material [11] - purports “broad agreement […that…] stigma surrounding opioid use is both significant and consequential” and has acted “as a barrier to reframe the opioid crisis as a public health issue”, concretely as it “affects how we conceptualize, frame and prioritize [the opioid] crisis.” Stigma is furthermore stated to lead to “hiding and creates barriers to help-seeking, [… and that it] contributes to ongoing system mistrust and avoidance of services […and…] results in poorer quality care and response”. For principal remedies, these campaign documents then – specifically also as actions geared towards “health leaders” - prescribe “comprehensive stigma reduction and intervention strategies for frontline providers”, “address[ing] the ethical dilemmas experienced by … front-line providers regarding high-recidivism clients and the emergency-relief measures (e.g., Narcan) that may increase risk-behaviors”; and “increas[ing] the use of non-stigmatising language and establish[ing] best practice guidelines for opioid-related terminology and language”; ensuring “stigma-informed … prevention and policies efforts”; and removal of “organizational and policy-related barriers … to a full range of care interventions and services”. Yet here also, it is then acknowledged that "the evidence base supporting anti-stigma interventions in this [opioid] area is thin, and first requires a “more robust body of evidence”. In related media release-statements, the CPHO publicly called on “health leaders to tackle stigma [and] that we are all responsible for stopping it” [5], and the MHCC’s CEO declares that “naming stigma as a public health crisis is brave, bold and necessary.” [12]

One could be left with the distinct impressions, based on the above documents and statements, that the opioid crisis and its grave health and social toll in Canada are primarily a product of forces of ‘stigma’, and that implementing the suggested remedies will reliably guide and bring about much awaited, tangible improvement and solutions. While ‘stigma’ is a certainly present phenomenon and social dynamic in the substance use realm, and adversely affects substance users’ lives, behaviors and care in a multitude of ways, the above observations and remedies appear to be problematically narrow if not simplistic at key ends, while selective and featuring essential gaps in key elements and elaborations – especially also when considering their originating sources. These impressions sit uneasily, and warrant some basic while subjective consideration and comments as per the following brief summaries of main illustrative examples:

1. An (if not the) essential root driver and determinant of ‘stigmatization’, or the enactment of a fundamentally divisive ‘us versus them’ disposition for psychoactive substance users – for example, opioid users in the specific context of the opioid crisis, but beyond involving other illicit substances in other contexts -- is the fact that such use is categorically defined as a (criminally) illegal. This is so the case in Canada specifically per definition of the *Controlled Drugs and Substance Act (CDSA)*. Arguably, there is no more powerful and impactful social tool to create, and project stigma on a structural level, and its direct and indirect adverse consequences, than through criminalizing a specific behavior and the people such criminalization identifies and targets [13]. The criminal law, by definition, non-negotiably defines and enshrines most fundamental and shared social norms and values, and identifies actions and behaviors that violate and harm the social body of common rules which are then prescribed punishment as the state’s most powerful form of power [14]. The criminal law, therefore constitutes the authoritative, state-sanctioned basis and seal of stigma in the context of a law-based society: for primary examples ‘murder’, ‘treason’ or ‘assault’ carry irrevocable, heavy, official ‘stigma’ for the law-breaking act, and those who commit it. Moreover, the criminal law, or the process of crminalization, defines socially harmful and shunned actions, both by official and formal definition and its every-day enactment (e.g., enforcement); additionally, criminalization serves as the ultimately legitimate reference or justification for certain behaviors – or their ‘actors’ – to be differentiated, excluded, or penalized from many realms of life. Beyond, there is extensive scientific evidence on how ‘criminalization’ adversely affects substance use-related risks, harms and help seeking or service access [15–17]. Yet, nowhere do either report centrally name this quintessential link, or provide explicit recommendations that the ‘criminalization’ of drug use as a root driver of ‘stigma’ consequences ought to be materially corrected for the “cycle of stigma” to be slowed or stopped. This is particularly surprising since both reports come from leading federal government (CPHO) or arms’ length (MHCC) entities located at the very jurisdictional level of the CDSA as federal law in Canada. Both entities would be in a preeminent position to recognize, and emphasize for the explicit criminalization of substance (opioid) use as a primary, fundamental foundation of stigma that requires revision in order for the desired ‘stigma reduction’ to occur.
2. Similarly, there are other concrete, major intervention and policy actions – or gaps, rather – towards opioid-related public health measures along the lines of barriers and obstacles mentioned in theory that, for long, have been resisted by the very anti-stigma campaign protagonists. Tangibly, federal government authorities, for considerable time, have refused to formally call a ‘public health emergency’ (under the *Emergencies Act* in Canada) in response to the opioid crisis that would have allowed considerably more flexible and substantive measures to address and reduce related health risks and adverse outcomes, including the massive overdose mortality toll [18, 19]. Related, federal authorities have long resisted the implementation of broad-based, systematic provisions and measures for comprehensive ‘safer opioid’ distribution programming towards better protecting the numerous ‘at-risk’ opioid users from increasing exposure to highly toxic/potent, illicit opioid supply and elevated risk for overdose and death [20–22]. Both types of measures reflect and mimic standard interventions applied and enacted elsewhere (e.g., for vaccinations for influenza, or acutely extensive transmission control measures COVID-19 etc. [23]). On this basis, seemingly, preeminent health leaders emphasizing the exceptional burden from and need to “end the stigma cycle” themselves appear to be hindered or hesitant in their own efforts by ‘stigma-related policy barriers’ with substantial room for change towards more determined, concrete action.
3. The respective CPHO and MHCC reports appear to present a questionably one-directional or simplistic perspective on the mechanics and nature of ‘stigma’. While the reports’ analyses suggest multiple ‘pathways’ or ‘layers’ of stigma, they essentially appear to suggest that ‘stigma’ is an exclusively negative force in functioning and outcomes, and brings on extensive harm in whatever it touches or affects; therefore, any available measures ought to be deployed for it to be ‘purged’ for its major, consequential harms to be reduced in desirable ways for general benefit. But the dynamics and workings of ‘stigma’, if considered more fulsomely, are much more complex, or multi-dimensional. For example, while surely there should not be genuine intent to negatively label, or categorically stereotype, drug users as ‘bad persons’, there are many behaviors – in the social realities of daily life, health, or substance use – that are widely recognized and agreed as factually unhealthy and undesirable, and therefore feature legitimate reason (e.g., for the benefit of interventions) to be negatively labelled. For example, ‘drinking and driving’, smoking in front of children, sharing injection paraphernalia, or stealing are generally agreed-upon risky or harmful behaviors which are – for education or prevention messaging or deterrence purposes – negatively labelled and so conveyed for arguably good reason [24, 25]. It cannot be in anyone’s (and especially not prominent health leaders’) real interest to suggest correction of these behaviors to positive, or even neutral status or messaging in the interest of all-encomassing ‘stigma reduction’. Rather, the real, while presumably more complex challenge for a meaningful addressing of stigma appears to be, as far as possible, to dis-associate concrete, while undesirable or shunned behaviors and their negative labels from the general identity of those individuals or human beings who engage in or are associated with them, or least better consider and contextualize their real-life contexts, but not have their social or health or other existential opportunities categorically labelled or negatively burdened by them [10, 26]. This separation of value attributions is not an easy task, also given that negative behaviors do not exist without people associated with them, and one that will likely never be perfectly possible; however, to imply or invoke a functioning social world in which everything and anything will be free of negative labels – i.e., without any negative association or labels – is neither meaningfully possible nor workable.
4. Specifically in the realm of the opioid crisis and its distinct evolution, there have been other, powerful forces of social ‘labelling’ or associations at work that may be (perhaps somewhat clumsily) referred to as ‘false’ or ‘reverse stigma’ (i.e., suggesting misguided positive signals or properties on behaviors where the opposite, or at least active prudence or caution would have been warranted); these can be assumed to have led to at least as much, but likely far more, opioid-harms in the population as the genuine (adverse) ‘stigma’ drivers and effects laid out in the reports. As a key example, an essential causal driver of the opioid crisis, specifically as it unfolded in North America, has included the widespread, excessive medical availability, prescription and usage of potent opioid medications starting in the early 2000s. The vast increases in general population-wide opioid use, initially under the premise of improved population-wide pain care and featuring new - supposedly effective and side effect-free - opioid medications (e.g., oxycodone) aggressively promoted by pharmaceutical companies and facilitated by skewed prescription guidelines, insufficient regulation and prescriber practices alike, pushed large sub-populations into hazardous trajectories of opioid use, with many resulting in misuse, dependence or overdose deaths [27–29]. Later opioid formulations – incorrectly – were claimed to be ‘abuse-deterrent’ or ‘tamper-resistant’, and therefore safe from harm [30, 31]. All this related to government-licensed and -approved drugs, and occurred under the knowing eye of government monitoring and regulatory control. For (too) long, key government and regulatory authorities provided no relevant policy responses, and then did ‘too little too late’, to stop the detrimental dynamics of the opioid crisis and its massive population health harms unfolding in slow-motion [28]. As much as negative ‘stigma’ may push some opioid users into riskier behaviors or contribute to help or service access barriers and inferior care quality, as much did misleadingly, or simply false systemic positive social messages, images and pervasive assumptions about opioid medications and their alleged benefits, and related harmful (e.g., over-prescribing) practices endorsed or tolerated by key authorities contribute to the present opioid public health crisis. (Notably, one of the reports considered points out that users of ‘prescription opioids’ “also” experienced negative stigmatization, making users feel “addicted … as much as a heroin addict” – implying a needed differentiation in stigma attribution between users of 'medical' and 'non-medical' drugs) ([6], p.44). These above factors ought to be taken into account especially when examining ‘stigma’ as one form of a social process influencing behaviors and adverse outcomes, whereas these are complemented by other social processes leading to and contributing to the same problem's formation and consequences.
5. There is a sizeable, recent body of scientific literature – e.g., specifically including systematic reviews from the past decade – devoted to stigma, substance use and related interventions. Surprisingly, little of these evidence-based insights appear to be considered in comprehensive depth in either of the two documents. For example, while respective reviews find ample evidence for a common presence of negative attitudes and beliefs towards substance users among health professionals or policy representatives, and correspondingly ample accounts of such experiences and consequences among substance users themselves, other key elements of empirical knowledge on or understanding of ‘stigma’, and especially effective counter-actions appear to be much more restricted [32–34]. Concretely, there is a lack of essential construct, measurement and definitional clarity and consistency, and a dearth of rigorous (e.g., longitudinal) studies and other research on stigma [32, 35]; there overall are few consistent findings on the relationship between stigma and substance use, and few studies have evaluated actual consequences of subjective ‘stigma’ impressions [36]; and evidence on effective stigma-reducing interventions is considered limited [37]. Crapanzano et al. (2014) report the notable finding in their (medical student) study sample that these believed that stigma beliefs among health care professionals were indeed common, but that their own beliefs and care practices would not be influenced by these [38].

## Conclusions

There appears to be good reason for some sensible reflection or restraint to be applied on the above stigma-fighting campaign and action front, specifically as generously projected on the ‘opioid crisis’ in Canada. There is little doubt that ample stigmatizing forces and experiences exist and crucially work against the health and wellbeing of substance users in many ways, and should be tackled and alleviated. To which extent this can be most effectively achieved mostly by ‘language adjustments’, ‘resilience strengthening’ or similar efforts suggested, everyone may consider and guess for themselves – also since current scientific knowledge does not provide much conceptual clarity or substantive evidence what such efforts tangible mean or can accomplish in material reality. The dynamics and effects of stigma for substance use, and both meaningful and realistic ways towards addressing and working to resolve these, however, can be assumed to be much more complex and challenging than what the above-cited two documents and their - rather narrow, if not simplistic - accounts suggest. They may be so described, since they present only limited insights on the (e.g., structural, social and individual) causes or drivers of stigma for substance use, and possible promising and effective remedies for material and sustained change in the lives of those concerned (i.e., substance users). These factors require, and deserve, deeper and better examination and analysis for realistic contributions and improvements for the important stigma-related causes and issues at hand – especially from the leading and privileged authorities from which have put forward these reports. First and foremost, the quintessential causal role of the criminalization of illicit substance use (and thereby its users) for the pervasive production of structural stigma needs to feature prominent recognition, and related calls for change in such a campaign if sincerely committed to effective and material stigma reduction.

There appear, however, a couple of other latent risks or adverse effects associated with this kind of ‘*en passant*’-kind of ‘anti-stigma’ presentation and campaigning that avoids to name core causes and elements. One is that it can be dangerously seductive as a self-righteous, or serving platform on which now ‘stigma’ is staged as a convenient, general or principal ‘scapegoat’ for the opioid crisis, and its ongoing massive and persistent harms. Calling out, rejecting and fighting ‘stigma’ as a socially shared villainous force – akin, for example, to N. Christie’s ‘suitable enemy’ concept for illicit drugs [39] – is somewhat similar to promoting ‘motherhood and apple-pie’ (or supporting justice, equality, and peace for all), while rather limited in applied value or impact if mainly remaining at rhetorical or symbolic levels, and not realistically translated into necessary material action or change at the causal foundations. The other is that such social campaigns may (too easily) serve as a distraction from those tangible or structural actions or measures urgently required to improve and protect the existential real-life conditions, and elementary health and wellbeing (including basic, daily survival through effective, comprehensive overdose prevention services) of the many at-risk opioid (or all substance) users. The current, long-lasting fight against the opioid public health crisis will not be won by campaigns against stigma in itself. Rather, fundamental drug law and policy reform, i.e. purging the intent criminalization (and related material stigmatization) of drug use/possession as a ‘criminal act’, and consequentially defining ‘the user’ as a criminal being with all adverse consequences – including fundamentally negative stigma – that entails is a (the) foremost action priority for this end [40]. Much of this, if materially enacted, will provide and bring fundamentally ‘de-stigmatizing’ effects for substance users in many crucial (direct and indirect) ways. No such measures, however, are clearly laid out in the report documents mentioned, and thus form quintessential gaps towards substantive and effective anti-stigma efforts in this realm.

Concretely, after > 25,000 opioid-related deaths in merely a decade, Canada yet in 2020 lacks essential elements of a comprehensive, consistent and committed ‘public health emergency’ strategy, and essential public health interventions, including reliable, national ‘safer opioid distribution’ provisions, for at-risk opioid users. It is when these urgent, material remedy needs and action gaps are effectively addressed by the health and policy leaders in charge, we should devote resources to an improved, in-depth understanding and effective addressing of what may be the remaining elements of stigma in the real of substance use, and the people who use them.

## Data Availability

Not applicable.

## Abbreviations

QOL: Quality of Life
MHCC: Mental Health Commission of Canada
CPHO: Chief Public Health Officer of Canada
CDSA: Controlled Drugs and Substances Act
